# Efficacy and safety of PD-1/PD-L1 immune checkpoint inhibitors in the treatment of recurrent ovarian cancer: A systematic review and meta-analysis

**DOI:** 10.1097/MD.0000000000038019

**Published:** 2024-05-03

**Authors:** Yafang Chen, Xiaomei Liu, Ying Hu, Lingling Xia

**Affiliations:** a Oncology Department Three Wards, The First Affiliated Hospital of Army Medical University, Chongqing, China.

**Keywords:** immune checkpoint inhibitors, meta-analysis, PD-1 inhibitors, PD-L1 inhibitors, recurrent ovarian cancer

## Abstract

**Background:**

Recurrent ovarian cancer (OC) presents a significant therapeutic challenge with limited treatment success. Programmed cell death protein 1 (PD-1/PD-L1) immune checkpoint inhibitors have emerged as a potential treatment avenue, necessitating a systematic review and meta-analysis to evaluate their efficacy and safety.

**Methods:**

Adhering to preferred reporting items for systematic reviews and meta-analyses guidelines, we conducted a comprehensive literature search across PubMed, Embase, Web of Science, and Cochrane Library, culminating in the inclusion of studies focusing on the treatment of recurrent OC with PD-1/PD-L1 inhibitors. Studies were evaluated using the Newcastle-Ottawa Scale and analyzed using fixed or random effects models depending on heterogeneity levels.

**Results:**

Our search yielded 1215 articles, with 6 meeting the inclusion criteria for final analysis. Studies varied in size and reported median age, overall survival (OS), progression-free survival (PFS), and adverse events. The meta-analysis showed improved Objective Response Rates (ORR), Disease Control Rate (DCR), and PFS in patients treated with PD-1/PD-L1 inhibitors. The overall adverse event rate was 17.9%, indicating a need for careful patient selection and monitoring. No significant publication bias was detected, enhancing the reliability of our findings.

**Conclusions:**

PD-1/PD-L1 inhibitors offer a promising treatment option for recurrent OC, improving ORR, DCR, and PFS. However, the higher incidence of adverse events necessitates a cautious approach to their use. Future research should focus on long-term outcomes, biomarker identification, and optimal combination therapies.

## 1. Introduction

Ovarian cancer (OC) is the most lethal gynecologic malignancy, characterized by late-stage presentation and a high propensity for recurrence.^[1]^ The majority of patients are diagnosed at an advanced stage, leading to a poor prognosis and limited treatment options.^[2]^ Standard treatment modalities, including surgery, chemotherapy, and radiation therapy, have provided some improvements in patient outcomes, but the recurrence rate remains high, and survival rates have not significantly improved over the past decades.^[3,4]^ Recent advancements in immunotherapy, particularly the development of immune checkpoint inhibitors targeting the PD-1/PD-L1 axis, have shown promise in a variety of cancers, including melanoma, non-small cell lung cancer, and renal cell carcinoma.^[5]^ These agents work by inhibiting the interaction between the programmed cell death protein 1 (PD-1) on T-cells and its ligand PD-L1 on tumor cells, thereby enhancing the immune system response against the tumor.^[6]^

The expression of PD-L1 on tumor cells and infiltrating immune cells in the OC microenvironment has been documented, suggesting a rationale for exploring PD-1/PD-L1 inhibitors as a therapeutic option in recurrent OC.^[7,8]^ However, the therapeutic efficacy and safety profile of these inhibitors in OC patients need systematic evaluation due to the variability in response and potential adverse effects associated with immunotherapy.^[9,10]^ Several studies and clinical trials have investigated the use of PD-1/PD-L1 inhibitors in OC, with outcomes ranging from significant response to limited activity depending on various factors such as tumor PD-L1 expression, the tumor microenvironment, and previous treatment history.^[11]^ The results are promising yet inconclusive, necessitating a comprehensive analysis to understand the overall benefit and risk profile of these agents in recurrent OC.

By providing a rigorous and comprehensive analysis of the available data, this study seeks to clarify the role of PD-1/PD-L1 inhibitors in the treatment paradigm of recurrent OC and assist in clinical decision-making. It is intended to contribute to a better understanding of the potential benefits and risks associated with these therapies, thereby informing future research and practice in this challenging clinical context.

## 2. Materials and methods

### 2.1. Search strategy for meta-analysis

In conducting the meta-analysis, the research team adhered to the preferred reporting items for systematic reviews and meta-analyses guidelines.^[12]^ The literature search was completed using 4 electronic databases: PubMed, Embase, Web of Science, and Cochrane Library, on September 19, 2023, without imposing any time restrictions. Key terms employed in the search included “ovarian cancer,” “pd-1 inhibitors,” “pd-l1 inhibitors,” “immune checkpoint inhibitors,” and “immunotherapy.” These terms were meticulously chosen to cover the extensive range of the patient, intervention, comparison, outcome framework, aiming for a thorough collection of pertinent studies for the meta-analysis. There were no restrictions based on language. Additionally, reference lists from identified articles were manually examined to uncover any further relevant publications. This comprehensive search strategy was designed to encompass a wide array of studies, ensuring a detailed and exhaustive meta-analysis.

### 2.2. Inclusion criteria and exclusion criteria

The meta-analysis included studies that were focused on patients diagnosed with recurrent OC treated with PD-1/PD-L1 immune checkpoint inhibitors. Included studies had to report on the efficacy and safety outcomes of the treatment, such as overall survival (OS), progression-free survival (PFS), response rate, and adverse events. The analysis considered randomized controlled trials, cohort studies, case-control studies, and case series that provided clear data on treatment effects. Studies were required to be published in peer-reviewed journals and could be of any language, provided that an English abstract was available.

Excluded from the meta-analysis were studies that did not specifically address recurrent OC or did not focus on PD-1/PD-L1 immune checkpoint inhibitors as a primary treatment modality. Studies were also excluded if they were abstracts, conference presentations, editorials, or letters without sufficient data. Preclinical studies, animal studies, and studies without accessible full-text articles were also omitted. Furthermore, studies that did not provide specific data on treatment outcomes relevant to the efficacy or safety of PD-1/PD-L1 inhibitors, or those that had overlapping data previously published in other studies (to prevent double counting), were not considered. Any studies with significant methodological flaws or incomplete data were excluded to ensure the quality and reliability of the meta-analysis findings.

### 2.3. Data extraction

The data extraction process was conducted systematically and independently by 2 reviewers, with any arising discrepancies resolved through discussion or, if necessary, arbitration by a third reviewer. The extracted data encompassed key study characteristics and metrics pertinent to assessing the efficacy and safety of PD-1/PD-L1 immune checkpoint inhibitors in the treatment of recurrent OC. Specifically, the following details were extracted from each selected study: author names, publication year, study design, total number of participants and the number in each study group, incidence of adverse events, and median age of patients in the treatment group. In terms of efficacy and safety outcomes, the data included OS rates, PFS, objective response rates (ORR), and disease control rates (DCR). In instances where relevant data were missing or incomplete in the published reports, attempts were made to contact the original investigators via email to request any unpublished data or additional information required for the comprehensive analysis.

### 2.4. Quality assessment

In evaluating the quality of studies for our meta-analysis, each was appraised independently by 2 reviewers using the Newcastle-Ottawa Scale (NOS).^[13]^ This scale is a renowned assessment tool, dividing the quality criteria into 3 main domains: selection, comparability, and outcome, which helps identify potential biases within the studies. Each study received a score out of a total of 9, reflecting its overall quality and rigor. Studies were then classified based on their scores: a total of 0 to 3 indicated low quality, 4 to 6 suggested moderate quality, and 7 to 9 represented high-quality studies. This systematic scoring and categorization ensured a standardized and comprehensive quality assessment across all included studies.

### 2.5. Statistical analyses

In the meta-analysis, the statistical analyses were designed and executed to assess study heterogeneity, effect size, potential biases, and overall robustness of findings. The heterogeneity among included studies was first evaluated using chi-square statistics and quantified by the *I*^2^ value. A low *I*^2^ value (<50%) and a *P* value of .10 or higher indicated a lack of significant heterogeneity, prompting the use of a fixed-effect model to calculate the combined effect size. Conversely, significant heterogeneity was suggested by an *I*^2^ value of 50% or higher, or a *P* value <.10, in which case a random effects model was employed for effect size calculation. Sensitivity analysis played a critical role in verifying the stability of the results and identifying potential sources of heterogeneity. This involved sequentially omitting each study to observe the impact on the overall effect size. Additionally, publication bias was assessed both visually through the symmetry of funnel plots and quantitatively using Egger linear regression test. An asymmetrical funnel plot or a significant Egger test indicated potential publication bias. All statistical tests were 2-sided, with a significance threshold set at a *P* value < .05. Data were rigorously analyzed using Stata version 17, ensuring a comprehensive and reliable statistical evaluation of the included studies.

## 3. Results

### 3.1. Search results and study selection

**Figure 1.**
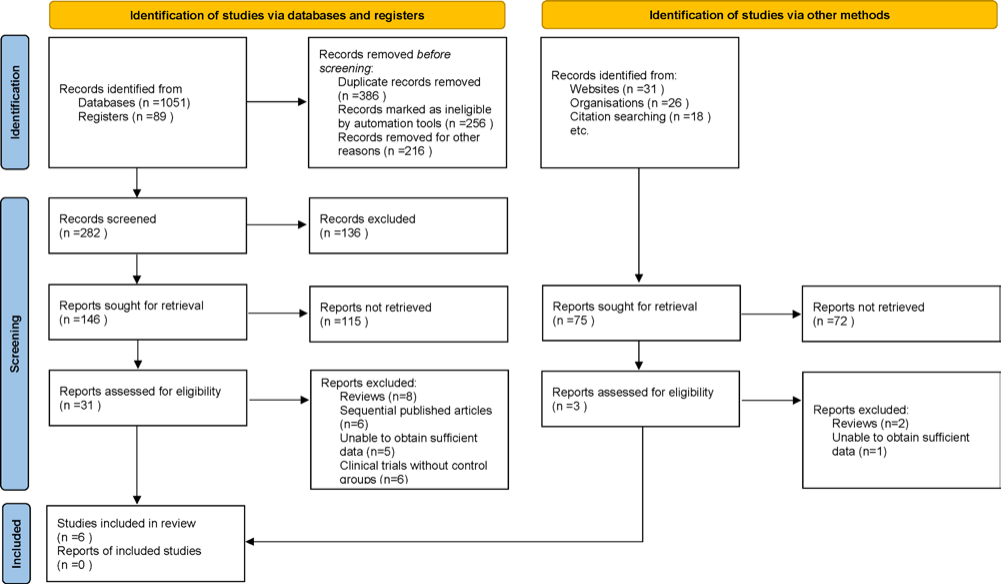
Study selection flowchart for meta-analysis inclusion.

In this systematic review and meta-analysis, an initial search in various databases yielded 1215 potentially relevant articles. After removing duplicates and rigorously screening titles and abstracts based on defined criteria, 34 articles remained. These underwent full-text review by multiple investigators, leading to the exclusion of 28 due to reasons like being review articles, having insufficient data, or lacking necessary control groups. Ultimately, 6 studies met all inclusion criteria and were selected for the final meta-analysis.^[14–19]^ This process ensured a focused and high-quality pool of research for comprehensive synthesis and analysis (Fig. 1).

### 3.2. Study characteristics

The meta-analysis synthesized data from various studies focusing on the treatment of recurrent OC using PD-1/PD-L1 inhibitors. The selected studies varied in sample size, ranging from smaller cohorts of 17 patients to larger groups of up to 285 patients, indicating a diverse set of data. The median age of participants across these studies, when reported, typically hovered in the early 60s, reflecting the demographic most affected by recurrent OC. However, not all studies provided specific information on median age, OS, or PFS. The treatments evaluated across these studies included a range of PD-1/PD-L1 inhibitors, such as BMS-936559, Tislelizumab, Nivolumab, and Pembrolizumab. While several studies did not report median OS, they provided valuable data on median PFS, with durations ranging from a few weeks to several months. This variety in reported outcomes demonstrates the heterogeneity in response to PD-1/PD-L1 inhibitors, as well as the variance in reporting practices across different studies (Table 1).

**Table 1:** Summary of studies included in the meta-analysis.

Author	Yr	Sample size	Median age	Median OS	Median PFS	Treatment
Brahmer et al^[14]^	2012	17	NA	NA	NA	BMS-936559
Desai et al^[15]^	2020	51	61	NA	NA	Tislelizumab
Hamanishi et al^[16]^	2015	20	60	NA	3.5 mo	Nivolumab
Liao et al^[17]^	2019	27	64	NA	2.6 mo	Pembrolizumab
Marius et al^[18]^	2019	18	61	30 wk	15 wk	Nivolumab
Matulonis1 et al^[19]^	2019	285	NA	Not reached	2.1 mo	Pembrolizumab

NA = not available, OS = overall survival, PFS = progression-free survival.

### 3.3. Results of quality assessment

In the quality assessment conducted using the NOS, each study was evaluated on several criteria including representativeness of the exposed cohort, selection of the non-exposed cohort, ascertainment of exposure, initial absence of the outcome, comparability of cohorts, assessment of outcome, duration of follow-up, and adequacy of cohort follow-up. The studies scored between 7 and 9 out of a maximum of 9 points, reflecting a range from good to high-quality. Specifically, 1 study scored 7 points, indicating good quality with potential for improvement in certain areas. Two studies scored 8 points, showing very good quality with most criteria well addressed. Three studies achieved the highest score of 9 points, reflecting excellent study quality across all evaluated criteria (Table 2).

**Table 2:** The quality assessment according to Newcastle-Ottawa Scale of each cohort study.

Study	Representativeness of the exposed cohort	Selection of the non-exposed cohort	Ascertainment of exposure	Demonstration that outcome of interest was not present at start of study	Comparability of cohorts on the basis of the design or analysis	Assessment of outcome	Was follow-up long enough	Adequacy of follow-up of cohorts	Total score
Brahmer et al^[14]^	★	★	★	★	★★	★		★	8
Desai et al^[15]^	★	★	★	★	★★	★	★	★	9
Hamanishi et al^[16]^	★	★	★	★	★	★	★	★	8
Liao et al^[17]^	★		★	★	★	★	★	★	7
Marius et al^[18]^	★	★	★	★	★★	★	★	★	9
Matulonis1 et al^[19]^	★	★	★	★	★★	★	★	★	9

★: each individual asterisk (“★”) signifies one point.

### 3.4. Meta-analytic evaluation of PD-1/PD-L1 inhibitors on ORRs in recurrent OC treatment

**Figure 2.**
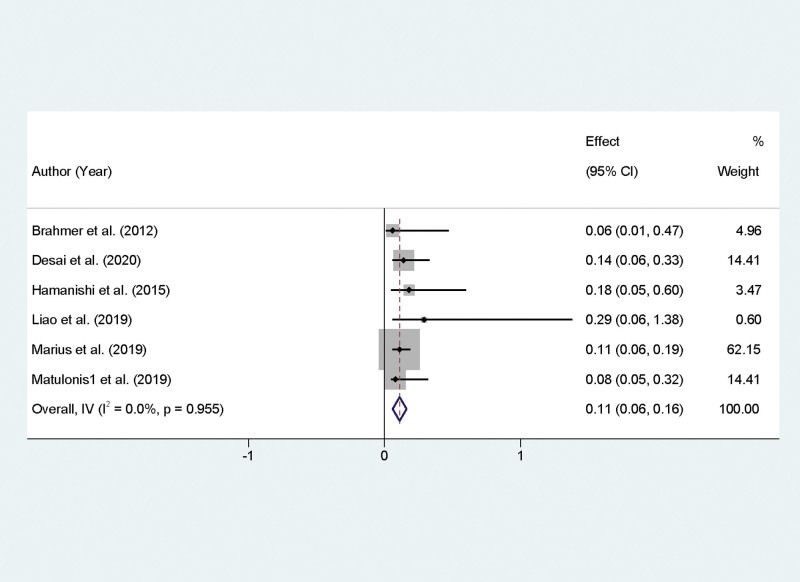
Forest plot illustrating objective response rates for PD-1/PD-L1 inhibitors in recurrent ovarian cancer treatment. PD-1/PD-L1 = programmed cell death protein 1.

In assessing the efficacy of PD-1/PD-L1 inhibitors for the treatment of recurrent OC, our meta-analysis incorporated data on ORR from 6 studies. A thorough analysis of 6 studies revealed no significant heterogeneity (*P* = .955, *I*² = 0%), allowing for the employment of a fixed-effect model for effect size calculation. The meta-analytic synthesis yielded a combined effect size (odds ratio, OR) of 0.11 with a 95% CI of 0.06 to 0.16, which was statistically significant (*P* < .01). Upon statistical transformation, the derived effect size corresponds to an aggregated ORR of 11.6%, with the 95% confidence interval spanning from 6.9% to 12.5%, for patients treated with PD-1/PD-L1 inhibitors (Fig. 2).

### 3.5. Meta-analysis of DCR in recurrent OC managed with PD-1/PD-L1 inhibitors

**Figure 3.**
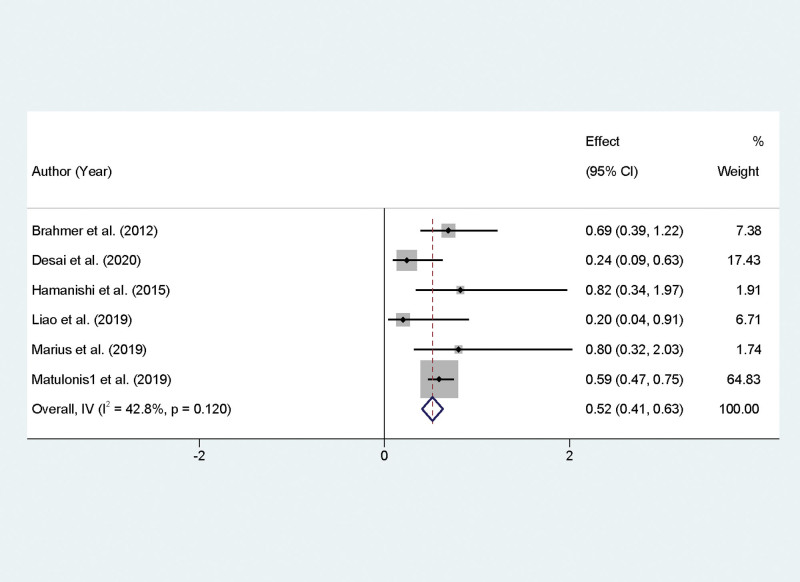
Forest plot demonstrating disease control rates in recurrent ovarian cancer treated with PD-1/PD-L1 inhibitors. PD-1/PD-L1 = programmed cell death protein 1.

Our meta-analysis focused on the DCR as an indicator of the efficacy of PD-1/PD-L1 inhibitors in the treatment of recurrent, refractory OC. The analysis included studies that reported on DCR, revealing low to moderate heterogeneity among the results (*I*² = 42.8%, *P* = .12). This heterogeneity was deemed acceptable for the application of a fixed-effect model to calculate the combined effect size. The synthesis of the included studies produced a combined OR of 0.52 with a 95% CI of 0.41 to 0.63, demonstrating a statistically significant effect (*P* < .01). Through statistical conversion, this effect size translates to a pooled DCR of 38.6%, with a 95% confidence interval ranging from 31.3% to 56.5% for patients receiving PD-1/PD-L1 inhibitors (Fig. 3).

### 3.6. Meta-analysis of PFS in recurrent OC with PD-1/PD-l1 inhibitor therapy

**Figure 4.**
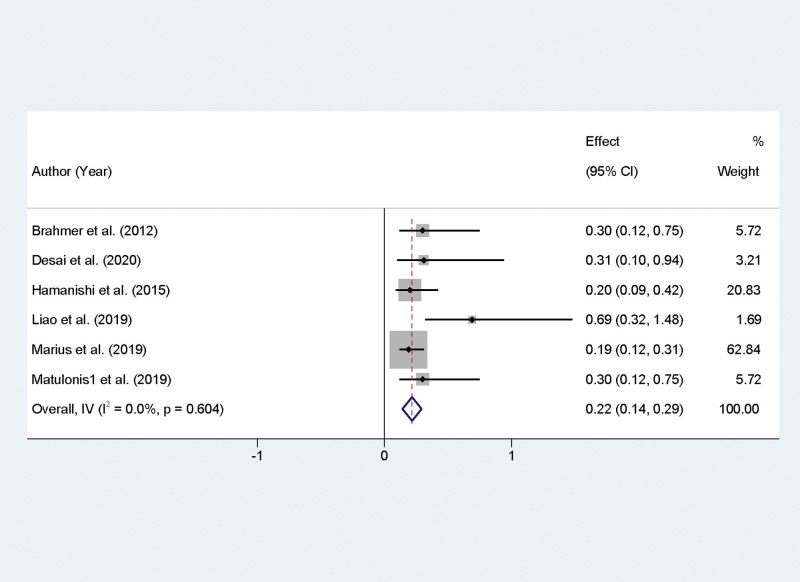
Forest plot representing progression-free survival rates in recurrent ovarian cancer treated with PD-1/PD-L1 inhibitors. PD-1/PD-L1 = programmed cell death protein 1.

In our meta-analysis, we evaluated the impact of PD-1/PD-L1 inhibitors on the PFS of patients with recurrent OC. The studies included in this analysis exhibited homogeneity in their results regarding PFS, as indicated by a zero percent *I*² statistic (*I*² = 0.0%, *P* = .604), which justifies the use of a fixed-effect model for the meta-analysis. The combined data from the included studies yielded an OR of 0.22, with a 95% CI of 0.14 to 0.29, and the result was statistically significant (*P* < .01). This OR was statistically converted into a pooled estimate of PFS, which was found to be 12.9%. The 95% confidence interval for this estimate ranged from 8.9% to 18.6%, indicating that PD-1/PD-L1 inhibitors provide a measurable benefit in terms of PFS for this patient population (Fig. 4).

### 3.7. Meta-analytic assessment of adverse event rates in recurrent OC patients receiving PD-1/PD-L1 inhibitor therapy

**Figure 5.**
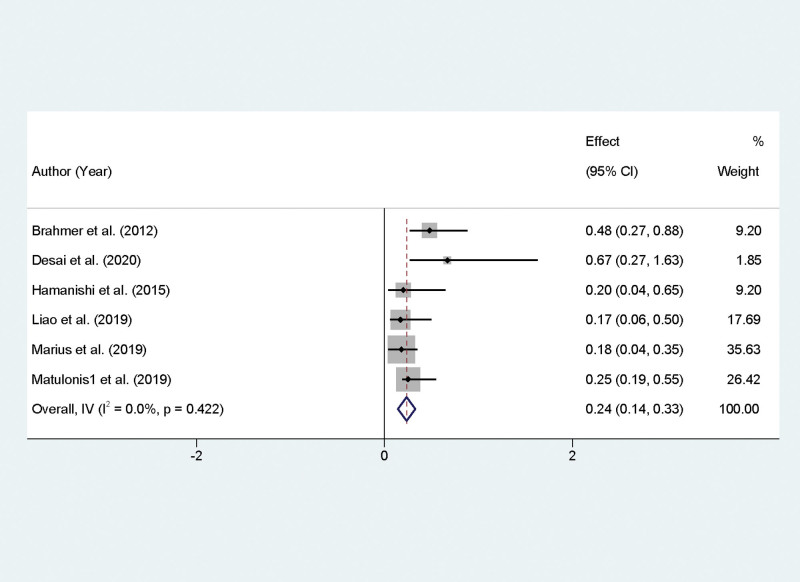
Forest plot depicting adverse event rates in recurrent ovarian cancer patients undergoing PD-1/PD-L1 inhibitor therapy. PD-1/PD-L1 = programmed cell death protein 1.

This meta-analysis rigorously evaluated the incidence of adverse events associated with PD-1/PD-L1 inhibitors in the treatment of recurrent OC. Analysis of the selected studies revealed a lack of heterogeneity concerning adverse events (*I*² = 0.0%, *P* = .422), supporting the use of a fixed-effect model for pooled data analysis. The synthesis of the studies provided a combined OR of 0.24 for the occurrence of adverse events, with a 95% CI of 0.14 to 0.33, indicating a statistically significant finding (*P* < .01). Statistical conversion of this OR elucidates that the estimated incidence rate of adverse events for patients under PD-1/PD-L1 inhibitor therapy is 17.9%, with the 95% confidence interval ranging from 11.96% to 28.1% (Fig. 5).

### 3.8. Publication bias assessment in meta-analysis of PD-1/PD-L1 inhibitors for recurrent OC

**Figure 6.**
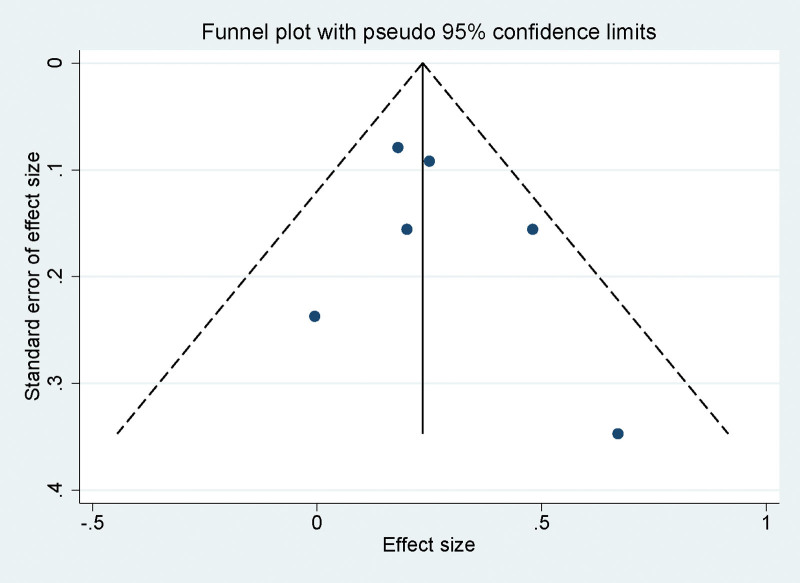
Funnel plot assessing publication bias in studies included in the meta-analysis.

To evaluate the presence of publication bias within our meta-analysis, funnel plots were generated for each set of pooled data. The symmetry observed in these plots suggests an absence of bias, with Figure 6 illustrating this balanced distribution. Complementing the visual assessment, Egger linear regression test was applied to the data across various variables. The test results did not show any significant publication bias (*P* > .05 for all variables), lending further support to the credibility of our meta-analytic findings.

## 4. Discussion

Our study enriches clinical decision-making in recurrent OC treatment by elaborating on the strategic use of PD-1/PD-L1 immune checkpoint inhibitors. It highlights their pivotal role in improving PFS and DCR, pivotal metrics in the management of this malignancy. Furthermore, our comprehensive assessment of adverse event rates assists oncologists in navigating the therapeutic landscape, weighing the benefits against potential risks. This nuanced understanding facilitates tailored treatment approaches, essential in a domain where the quest for efficacious and safe options remains critical.

In the treatment of recurrent OC, traditional approaches such as surgery, chemotherapy, and radiation have been the cornerstone; however, the prognosis remains poor due to high rates of recurrence and resistance to treatment.^[20,21]^ The advent of immune checkpoint inhibitors targeting the PD-1/PD-L1 axis has ushered in a new era of cancer immunotherapy, offering hope for improved outcomes in various malignancies, including recurrent OC.^[22]^ The rationale behind using PD-1/PD-L1 inhibitors is grounded in the immunosuppressive tumor microenvironment characteristic of OC, which allows tumor cells to evade immune detection and destruction.^[23,24]^ By blocking the interaction between PD-1 receptors on T-cells and PD-L1 on tumor cells, these agents reinvigorate the immune system ability to recognize and attack cancer cells.^[25,26]^ This systematic review and meta-analysis aimed to synthesize available evidence on the efficacy and safety of PD-1/PD-L1 inhibitors in recurrent OC, providing a comprehensive understanding of their therapeutic potential and limitations.

This meta-analysis provides a comprehensive overview of the efficacy and safety of PD-1/PD-L1 inhibitors in the treatment of recurrent OC, reflecting on various outcomes such as ORR, DCR, PFS, and adverse events. The findings reveal a nuanced landscape of immunotherapy role in managing this aggressive malignancy. The pooled data indicate a moderate ORR and DCR when patients are treated with PD-1/PD-L1 inhibitors. Specifically, an ORR of 11.6% and a DCR of 38.6% were observed. These rates, while not overwhelmingly high, are nonetheless significant for a population with limited treatment options and typically poor prognosis. The effectiveness of these inhibitors may be attributed to the restoration of the immune system ability to recognize and attack tumor cells by inhibiting the PD-1/PD-L1 pathway, which is often exploited by cancer cells for immune evasion. The variance in ORR and DCR might reflect the heterogeneous nature of OC, the differing expressions of PD-L1 across patient populations, or the varying lines of prior treatments received.

The analysis of PFS, which showed a pooled estimate of 12.9%, further supports the role of PD-1/PD-L1 inhibitors in delaying disease progression. This outcome is particularly encouraging, considering that recurrent OC is notoriously difficult to treat, and any extension in PFS can be clinically meaningful for patient quality of life and treatment planning. The underlying mechanism for the improvement in PFS is likely multifactorial, involving not only the direct antitumor effects of PD-1/PD-L1 blockade but also the modulation of the tumor microenvironment and the promotion of a more effective and sustained antitumor immune response. Adverse events associated with PD-1/PD-L1 inhibitors were also evaluated, with a pooled incidence rate of 17.9%. This relatively moderate rate of adverse events is consistent with the known safety profile of immunotherapies, which can include immune-related adverse effects but often are more tolerable than traditional chemotherapy. The management of these adverse events is crucial in clinical practice and requires vigilant monitoring and timely intervention. It also noteworthy that the lack of heterogeneity in adverse event rates suggests a consistent safety profile across the different studies and patient populations.

Our focus on PD-1/PD-L1 immune checkpoint inhibitors stems from their unique mechanism of action that reactivates the body immune response against tumor cells, a promising approach in the treatment of recurrent OC. Unlike traditional chemotherapy and targeted therapies, which directly attack cancer cells often with significant toxicity, PD-1/PD-L1 inhibitors offer a more targeted approach, potentially leading to better tolerability and prolonged disease control. This positions them as a significant addition to the oncological arsenal, particularly for patients who have exhausted other treatment options. Moreover, the potential for combination therapies involving PD-1/PD-L1 inhibitors and existing treatments, such as chemotherapy, targeted therapy, and radiation, represents a frontier in oncology research. Such combinations could leverage the strengths of each modality, enhancing efficacy while managing the adverse event profile. Current studies and clinical trials exploring these combinations have shown promising results, indicating that PD-1/PD-L1 inhibitors may not only serve as a standalone option but also significantly enhance the effectiveness of traditional therapies in a synergistic manner. Our analysis acknowledges the necessity of integrating PD-1/PD-L1 inhibitors within a broader therapeutic context, suggesting that their role in treating recurrent OC could be pivotal, especially as part of multidisciplinary treatment strategies. This approach aligns with the evolving landscape of oncology, where the goal is not only to extend life but also to improve the quality of life for patients facing this challenging disease.

The results of our publication bias assessment provide confidence in the robustness and reliability of our findings. The symmetrical funnel plots and non-significant Egger test indicate a low likelihood of publication bias influencing the results. This suggests that the literature included in this meta-analysis is representative of the available evidence and supports the validity of the conclusions drawn. The meta-analysis, while insightful, is subject to limitations such as heterogeneity in study designs and populations, which may impact the generalizability of results. Furthermore, the role of biomarkers like PD-L1 expression in predicting treatment response was inconsistently reported, necessitating future research to focus on identifying and validating these predictive markers. Additionally, most studies concentrated on short-term outcomes, underscoring the need for long-term follow-up to understand the sustained efficacy, survival benefits, and quality of life impacts of PD-1/PD-L1 inhibitors. Lastly, as cancer treatment paradigms shift toward combination therapies, future investigations should explore the potential of PD-1/PD-L1 inhibitors in conjunction with other treatments to enhance therapeutic outcomes for recurrent OC patients. These directions not only address current gaps but also pave the way for more personalized and effective treatment strategies.

Our study highlights the significance of PD-1/PD-L1 inhibitors in recurrent OC treatment, advocating for broader research into combination therapies and biomarker discovery. Future studies should investigate synergies between PD-1/PD-L1 inhibitors and novel agents like Glutaminase and PARP inhibitors, targeting both metabolic pathways and DNA repair mechanisms for enhanced efficacy and reduced resistance.^[27,28]^ The development of robust biomarkers for treatment response prediction is essential, encompassing genetic, epigenetic, and proteomic markers to refine patient selection and tailor treatment strategies.^[29]^ Additionally, cohort studies across diverse populations are crucial for addressing treatment disparities and improving outcomes.^[30,31]^ Ultimately, advancing OC treatment requires a multi-faceted approach, integrating innovative therapies, precision oncology, and comprehensive patient studies to personalize and improve care for this complex condition.

## 5. Conclusions

In treating recurrent OC, PD-1/PD-L1 immune checkpoint inhibitors have shown to improve ORRs, DCRs, and PFS. However, they are associated with a higher incidence of adverse events. Careful consideration of individual patient circumstances is crucial in employing these agents, balancing potential benefits with the risk of adverse effects for optimal patient outcomes.

## Author contributions

**Conceptualization:** Yafang Chen.

**Formal analysis:** Yafang Chen.

**Investigation:** Xiaomei Liu.

**Methodology:** Yafang Chen, Xiaomei Liu.

**Resources:** Ying Hu.

**Software:** Ying Hu.

**Supervision:** Lingling Xia.

**Visualization:** Lingling Xia.

**Writing – original draft:** Yafang Chen.

**Writing – review & editing:** Lingling Xia.

## References

[1] O’Malley DM. New therapies for ovarian cancer. J Natl Compr Canc Netw. 2019;17:619–21. 31117037 10.6004/jnccn.2019.5018

[2] Zsiros E Lynam S Attwood KM. Efficacy and safety of pembrolizumab in combination with bevacizumab and oral metronomic cyclophosphamide in the treatment of recurrent ovarian cancer: a phase 2 nonrandomized clinical trial. JAMA Oncol. 2021;7:78–85. 33211063 10.1001/jamaoncol.2020.5945 PMC7677872

[3] Filis P Mauri D Markozannes G. Hyperthermic intraperitoneal chemotherapy (HIPEC) for the management of primary advanced and recurrent ovarian cancer: a systematic review and meta-analysis of randomized trials. ESMO Open. 2022;7:100586. 36116421 10.1016/j.esmoop.2022.100586 PMC9588894

[4] Armstrong DK Alvarez RD Backes FJ. NCCN guidelines® insights: ovarian cancer, version 3.2022. J Natl Compr Canc Netw. 2022;20:972–80. 36075393 10.6004/jnccn.2022.0047

[5] Yang C Xia BR Zhang ZC. Immunotherapy for ovarian cancer: adjuvant, combination, and neoadjuvant. Front Immunol. 2020;11:577869. 33123161 10.3389/fimmu.2020.577869 PMC7572849

[6] Färkkilä A Gulhan DC Casado J. Immunogenomic profiling determines responses to combined PARP and PD-1 inhibition in ovarian cancer. Nat Commun. 2020;11:1459. 32193378 10.1038/s41467-020-15315-8 PMC7081234

[7] Pawłowska A Rekowska A Kuryło W. Current understanding on why ovarian cancer is resistant to immune checkpoint inhibitors. Int J Mol Sci. 2023;24:10859. 37446039 10.3390/ijms241310859 PMC10341806

[8] Alwosaibai K Aalmri S Mashhour M. PD-L1 is highly expressed in ovarian cancer and associated with cancer stem cells populations expressing CD44 and other stem cell markers. BMC Cancer. 2023;23:13. 36604635 10.1186/s12885-022-10404-x PMC9814309

[9] Dumitru A Dobrica EC Croitoru A. Focus on PD-1/PD-L1 as a therapeutic target in ovarian cancer. Int J Mol Sci. 2022;23:12067. 36292922 10.3390/ijms232012067 PMC9603705

[10] Peng H He X Wang Q. Immune checkpoint blockades in gynecological cancers: a review of clinical trials. Acta Obstet Gynecol Scand. 2022;101:941–51. 35751489 10.1111/aogs.14412 PMC9564814

[11] Zamarin D Burger RA Sill MW. Randomized phase II trial of nivolumab versus nivolumab and ipilimumab for recurrent or persistent ovarian cancer: an NRG oncology study. J Clin Oncol. 2020;38:1814–23. 32275468 10.1200/JCO.19.02059 PMC7255977

[12] Page MJ McKenzie JE Bossuyt PM. The PRISMA 2020 statement: an updated guideline for reporting systematic reviews. BMJ. 2021;372:n71. 33782057 10.1136/bmj.n71 PMC8005924

[13] Wells G. The Newcastle-Ottawa Scale (NOS) for assessing the quality of non-randomised studies in meta-analyses. 2014.

[14] Brahmer JR Tykodi SS Chow LQ. Safety and activity of anti-PD-L1 antibody in patients with advanced cancer. N Engl J Med. 2012;366:2455–65. 22658128 10.1056/NEJMoa1200694 PMC3563263

[15] Desai J Deva S Lee JS. Phase IA/IB study of single-agent tislelizumab, an investigational anti-PD-1 antibody, in solid tumors. J ImmunoTher Cancer. 2020;8:e000453. 32540858 10.1136/jitc-2019-000453 PMC7295442

[16] Hamanishi J Mandai M Ikeda T. Safety and antitumor activity of Anti-PD-1 antibody, nivolumab, in patients with platinum-resistant ovarian cancer. J Clin Oncol. 2015;33:4015–22. 26351349 10.1200/JCO.2015.62.3397

[17] Liao JB Gwin WR Urban RR. Pembrolizumab with low-dose carboplatin for recurrent platinum-resistant ovarian, fallopian tube, and primary peritoneal cancer: survival and immune correlates. J ImmunoTher Cancer. 2021;9:e003122. 34531249 10.1136/jitc-2021-003122 PMC8449961

[18] Marcus L Lemery SJ Keegan P. FDA approval summary: pembrolizumab for the treatment of microsatellite instability-high solid tumors. Clin Cancer Res. 2019;25:3753–8. 30787022 10.1158/1078-0432.CCR-18-4070

[19] Matulonis UA Shapira-Frommer R Santin AD. Antitumor activity and safety of pembrolizumab in patients with advanced recurrent ovarian cancer: results from the phase II KEYNOTE-100 study. Ann Oncol. 2019;30:1080–7. 31046082 10.1093/annonc/mdz135

[20] Sehouli J Grabowski JP. Surgery in recurrent ovarian cancer. Cancer. 2019;125(Suppl 24):4598–601. 31967681 10.1002/cncr.32511

[21] Świderska J Kozłowski M Kwiatkowski S Cymbaluk-Płoska A. Immunotherapy of ovarian cancer with particular emphasis on the PD-1/PDL-1 as target points. Cancers (Basel). 2021;13:6063. 34885169 10.3390/cancers13236063 PMC8656861

[22] Lee SM Lee S Cho HW. Application of immune checkpoint inhibitors in gynecological cancers: what do gynecologists need to know before using immune checkpoint inhibitors? Int J Mol Sci. 2023;24:974. 36674491 10.3390/ijms24020974 PMC9865129

[23] Saglam O Conejo-Garcia J. PD-1/PD-L1 immune checkpoint inhibitors in advanced cervical cancer. Integr Cancer Sci Ther. 2018;5. 10.15761/ICST.1000272 PMC6016855 29955379

[24] Gadducci A Guerrieri ME. Immune checkpoint inhibitors in gynecological cancers: update of literature and perspectives of clinical research. Anticancer Res. 2017;37:5955–65. 29061774 10.21873/anticanres.12042

[25] Zeng S Liu D Yu Y. Efficacy and safety of PD-1/PD-L1 inhibitors in the treatment of recurrent and refractory ovarian cancer: a systematic review and a meta-analysis. Front Pharmacol. 2023;14:1111061. 36992842 10.3389/fphar.2023.1111061 PMC10042289

[26] Chin CD Fares CM Konecny GE. Biomarkers that may predict response to immunotherapy in ovarian malignancies. Curr Opin Obstet Gynecol. 2020;32:84–90. 31804230 10.1097/GCO.0000000000000596

[27] Luna P Acharya G Ochola D. Abstract 5496: Glutaminase inhibition induces replication stress in ovarian cancer cells and inhibition of replication checkpoint causes synthetic lethality. Cancer Res. 2023;83(7_Supplement):5496–5496.

[28] Acharya G Mani C Reedy MB. Abstract 2667: PARG inhibition augments CHK1 inhibitor-induced replication stress and synergistically kills ovarian cancer cells. Cancer Res. 2023;83(7_Supplement):2667–2667.

[29] Mani C Acharya G Saamarthy K. Racial differences in RAD51 expression are regulated by miRNA-214-5P and its inhibition synergizes with olaparib in triple-negative breast cancer. Breast Cancer Res. 2023;25:44. 37081516 10.1186/s13058-023-01615-6 PMC10120249

[30] Acharya G Mani C Manne U. Abstract C027: miRNA-214-5P regulates RAD51, a biomarker for aggressive disease and racial disparities in triple-negative breast cancer. Cancer Epidemiol Biomark Prev. 2023;32(1_Supplement):C027–C027.

[31] Acharya GN Mani C Manne U. Abstract PO-131: RAD51 is a biomarker for aggressive disease and racial disparities in triple-negative breast cancer. Cancer Epidemiol Biomark Prev. 2022;31(1_Supplement):PO-131–PO-131.

